# The association between fear of future workplace violence and depressive symptoms among nurses based on different experiences of workplace violence: a cross-sectional study

**DOI:** 10.1186/s12912-023-01265-1

**Published:** 2023-04-15

**Authors:** Chang Fu, Xiaoqin Lv, Xuedan Cui, Minxiang Huang, Fenglin Cao

**Affiliations:** 1 Department of Health Service and Management, School of Public Health and Management, Binzhou Medical University, Yantai, Shandong China; 2 Department of Hepatobiliary and Pancreatic Surgery, Binzhou Medical University Hospital, Binzhou, Shandong China; 3 Office of Physician Training, Yidu Central Hospital of Weifang, Weifang, Shandong China; 4 Department of Pediatric Surgery, Binzhou Medical University Hospital, No.661, 2nd Huanghe Road, Binzhou, 256603 Shandong China; 5 Department of Health Psychology, School of Nursing and Rehabilitation, Cheeloo College of Medicine, Shandong University, 44 Wenhuaxilu Rd, Jinan, 250012 Shandong China

**Keywords:** Nurse, Fear of future workplace violence, Depressive symptoms, Workplace violence, Mental health

## Abstract

**Background:**

Fear of future workplace violence (FFWV) has a negative impact on individuals’ health. However, no study has investigated the association between FFWV and depressive symptoms. Nurses with different experiences of workplace violence may have different levels of FFWV and differences in mental health. This study explored the association between FFWV and depressive symptoms among Chinese nurses with different experiences of workplace violence.

**Methods:**

A cross-sectional study was conducted involving 1888 Chinese nurses from 12 tertiary hospitals in Shandong Province. The Fear of Future Violence at Work scale was used to measure FFWV. Depressive symptoms were assessed using the 10-item Center for Epidemiologic Studies Depression scale. Multiple logistic regression analysis was used to examine the association between FFWV and depressive symptoms.

**Results:**

The prevalence of depressive symptoms was 45.9% (no aggression group: 24.3%; non-physical violence group: 46.1%; physical violence group: 63.7%), and 72.8% of nurses had high levels of fear of future workplace violence (no aggression group: 60.2%; non-physical violence group: 75.6%; physical violence group: 70.8%). Compared with low levels of FFWV, high levels of FFWV were associated with more depressive symptoms among nurses in the no aggression group (odds ratio [OR] = 3.269, 95% confidence interval [CI]: 1.102–9.695) and in the non-physical violence group (OR = 2.338, 95% CI: 1.385–3.945).

**Conclusion:**

Nurses who had experienced physical violence had the most depressive symptoms and nurses with experience of non-physical violence had the greatest FFWV. Our findings suggested that there was a significant association between FFWV and depressive symptoms among Chinese nurses in the no aggression and non-physical violence groups. Hospital administrators need to address FFWV needs when developing strategies to reduce depressive symptoms among nurses.

## Introduction

Nurses play an irreplaceable role in assisting diagnosis and treatment of diseases, saving lives, alleviating pain, and promoting rehabilitation. The quality of nurses’ work is directly related to the efficacy of treatments for patients. However, in recent years, nurses have become at increased risk of mental health problems; this issue has become a concern worldwide. Depression is considered a multi-problematic disorder that leads to impairment in interpersonal, social and occupational functioning [1]. Almarhapi reported that the prevalence of depression was 40.8% among nurses in Saudi Arabia [2]. Fang found that the prevalence of depressive symptoms was 57.2% among nurses in China [3], which was higher than that found among older persons (37.5%) [4]. Previous studies have found that depressive symptoms has serious negative effects on nurses’ quality of life [5], job satisfaction [3], and the quality of care they provide [1]. Therefore, reducing the prevalence of depressive symptoms among nurses has become an urgent task for hospital administrators.

In recent decades, workplace violence (WPV) against nurses has become a global issue. WPV can be defined as any physical assault, threatening behavior, or verbal abuse that occurs in a work setting [6, 7]. A systematic review reported that there is a high incidence of WPV against nurses worldwide [8]. The incidence of WPV against nurses is also very high in China. A meta-analysis revealed that 71% of Chinese nurses had experienced WPV [9]. Many previous studies have focused on the effects of WPV on nurses’ mental health [10, 11], with reports of a positive association between WPV and depressive symptoms among nurses [3, 12]. However, the negative impact of WPV on nurses may stem not only from direct experiences of violence but also from nurses’ fear of future workplace violence (FFWV) [13].

Fear is one of the basic human emotions; its utility is to enable one to protect oneself from danger [14]. A high incidence of WPV and an unsafe work environment have been found to lead nurses to develop FFWV [15]. FFWV is an emotional response by an individual to a perceived risk of victimization in relation to WPV [16]. Researchers have paid increasing attention to the association between FFWV and work performance [17, 18]. However, the relationship between FFWV and depressive symptoms among nurses has not been studied. Shifting the focus from actual experiences of violence to fear of future violence may help researchers understand the links between WPV and its long-term negative psychological aspects and health outcomes [19]. Therefore, it is critical to investigate the relationship between FFWV and depressive symptoms among nurses.

WPV often includes verbal violence (such as abuse, sarcasm, and humiliation), psychological violence (such as baseless charges or complaints, and slander), and physical violence (such as biting, pushing, and sexual assault) [20–22]. Different types of WPV may be associated with different levels of FFWV and have varying effects on nurses’ mental health [23]. In addition, not only have nurses who directly experience WPV been reported to be afraid of future WPV, but nurses who have indirectly experienced WPV (such as witnessing WPV or learning about WPV against medical workers through news reports) have also been found to be afraid of future WPV. Therefore, the association between FFWV and mental health among nurses without experience of WPV also requires investigation. Therefore, the aims of this study were to investigate (i) the prevalence of depressive symptoms and levels of FFWV among nurses with different experiences of WPV; and (ii) the associations between FFWV and depressive symptoms among Chinese nurses with different experiences of WPV. The results of this study may provide new insights and suggestions for hospital managers to reduce the prevalence of depressive symptoms among nurses.

## Materials and methods

### Study design and participants

From July 30 to September 30, 2020, a cross-sectional survey was conducted among Chinese nurses in tertiary hospitals in Shandong Province. Shandong Province is a typical province in China in terms of demographics, society, and culture [24]. This survey adopted a multi-stage random sampling method as described in the literature [25]. The inclusion criteria for participants comprised voluntary participation, being a registered nurse, currently working as a nurse, and having worked in their current hospital for at least one year. Exclusion criteria comprised nurses on vacation at the time of the study or engaged in continuing education in another hospital, and persons with severe mental or physical impairments that may have prevented them from participating.

Informed consent was obtained from all participants, whose anonymity was ensured, along with the confidentiality of their responses. A total of 1933 electronic questionnaires were recovered from 12 tertiary hospitals. After excluding questionnaires with missing data, 1888 questionnaires were included in the analysis, for an effective response rate of 97.7%.

### Measures

#### Depressive symptoms

Depressive symptoms were measured using the 10-item Center for Epidemiological Study Depression (CES-D 10) scale, which has been found to have high reliability and validity in the Chinese population [26]. The CES-D 10 assesses the extent to which an individual has experienced depressive symptoms in the previous week. Possible answers for each CES-D 10 item include rarely (less than 1 day), sometimes (1–2 days), occasionally (3–4 days), or most of the time (5–7 days). The total score of the CES-D 10 ranges from 0 to 30, with higher scores indicating worse depressive symptoms. Ten points were used as the cutoff point to identify individuals with depressive symptoms [27]. Cronbach’s alpha values for the CES-D 10 in this study were as follows: no aggression group, α = 0.693; non-physical violence group, α = 0.657; and physical violence group, α = 0.732.

#### Fear of future workplace violence

In this study, nurses’ FFWV was measured using the Fear of Future Violence at Work scale. The scale was translated into Chinese by Fu and has high reliability and validity [25]. The 12-item Fear of Future Violence at Work scale was used to assess respondents’ fear of being exposed to WPV in the following year. Answers to each item were scored on a 7-point Likert scale, ranging from 1 to 7 (1 = strongly disagree, 7 = strongly agree). Total scores on the Fear of Future Violence at Work scale range from 12 to 84. The total scores of the Fear of Future Violence at Work scale were divided into three groups: 12–36 (low), 37–60 (moderate), and 61–84 (high) [24]. In this study, Cronbach’s alpha values for the Fear of Future Violence at Work scale were as follows: whole sample, α = 0.974; no aggression group, α = 0.980; non-physical violence group, α = 0.970; physical violence group, α = 0.980.

#### Other variables

The basic demographic information of the nurses collected in the study consisted of age, gender, marital status, living status, education level, and monthly income. In terms of marital status, participants were divided into married or single. In terms of living status, they were divided into living alone or living with others. Educational background categories comprised junior college or less, bachelor’s degree, and master’s degree or above. Monthly income categories included < 5000, 5000–8000, and ≥ 8001 Chinese Yuan (CNY). Job characteristics consisted of the department, professional title, employment status, number of vacation days per year, and working hours per week. The departments were divided into internal medicine, surgery, obstetrics and gynecology, pediatrics, emergency department, and others. Professional titles were categorized as primary, intermediate, and senior. Employment type was categorized as formal or contract. The number of vacation days per year was categorized as 0, 1–14, 15–30, and > 30 days. Working hours per week were divided into ≤ 40, 41–50, 51–60, and > 60 h. The number of children of the participants was divided into three groups (0, 1, and ≥ 2). Participants were also asked, “In the past 12 months, have you experienced verbal violence, psychological violence, or physical violence in the workplace?” [28].

### Data analysis

IBM SPSS Version 20.0 was used to analyze the data. Descriptive statistics were generated with categorical variables described using percentages and were generated with continuous variables described using means and standard deviations. Based on the experience of workplace violence, we divided our sample into three groups: no aggression group (nurses who did not suffer from workplace violence), non-physical violence group (nurses who suffered from verbal violence/psychological violence in the workplace), and physical violence group (nurses who suffered from physical violence in the workplace) [26]. Between-group comparisons were performed using χ^2^ tests for categorical variables and using Kruskal–Wallis test for continuous variables. The different scores of depressive symptoms among nurses according to different levels of FFWV was analyzed using One-way ANOVA. Multiple logistic regression was used to investigate the association between FFWV and depressive symptoms among nurses of each group.

## Results

### Sample characteristics

Table 1 shows the characteristics of the study population. Among the 1888 nurses, 93.9% were female, and 63.4% were 31–50 years old. Furthermore, 75.3% had at least one child and 80.1% were married. Of the sample, 87.2% were not living alone and 87.9% had a bachelor’s degree. Those receiving a monthly income of 5,000–8,000 CYN comprised the largest group within this category, accounting for 45.0% of the sample. Employment in a surgery department was reported by 39.1% of the sample. Almost half of the nurses had a primary title (47.4%) or an intermediate professional title (48.4%), and over half of the nurses were contract employees (78.3%). Most (59.4%) nurses took 1–14 vacation days per year, and 68.0% worked 41–50 h per week. A large proportion (71.7%) had experienced non-physical WPV and 15% had experienced physical WPV. Nurses in the non-physical violence group had the highest level of FFWV (75.6%), and the physical violence group had the highest prevalence of depressive symptoms (63.7%).

**Table 1:** Characteristics of the study population

Characteristics	Total (*n* = 1888)	No aggression (*n* = 251)	Non-physical violence (*n* = 1353)	Physical violence (*n* = 284)	*χ*^*2*^	*P*
Age, (%)					18.711	**0.010**^a^
≤ 30	619(32.8)	102(40.6)	407(30.1)	110(38.7)		
31–50	1197(63.4)	136(54.2)	896(66.2)	165(58.1)		
> 50	72(3.8)	13(5.2)	50(3.7)	9(3.2)		
Sex, (%)					35.330	** < 0.001**^a^
Male	116(6.1)	20(8.0)	58(4.3)	38(13.4)		
Female	1772(93.9)	231(92.0)	1295(95.7)	246(86.6)		
Marital status, (%)					17.295	** < 0.001**^a^
Single	376(19.9)	71(28.3)	239(17.7)	66(23.2)		
Married	1512(80.1)	180(71.7)	1114(82.3)	218(76.8)		
Living condition, (%)					17.983	** < 0.001**^a^
Living alone	241(12.8)	45(17.9)	145(10.7)	51(18.0)		
Not living alone	1647(87.2)	206(82.1)	1208(89.3)	233(82.0)		
Educational level, (%)					8.727	0.068^a^
Junior college or less	138(7.3)	28(11.2)	93(6.9)	17(6.0)		
Bachelor	1659(87.9)	216(86.1)	1191(88.0)	252(88.7)		
Master or above	91(4.8)	7(2.8)	69(5.1)	15(5.3)		
Monthly income(CNY), (%)					10.900	**0.028**^a^
< 5000	332(17.6)	61(24.3)	218(16.1)	53(18.7)		
5000–8000	850(45.0)	107(42.6)	612(45.2)	131(46.1)		
≥ 8001	706(37.4)	83(33.1)	523(38.7)	100(35.2)		
Number of children, (%)					15.435	**0.004**^a^
0	466(24.7)	82(32.7)	303(22.4)	81(28.5)		
1	902(47.8)	103(41.0)	673(49.7)	126(44.4)		
≥ 2	520(27.5)	66(26.3)	377(27.9)	77(27.1)		
Department					41.908	** < 0.001**^a^
Internal medicine	535(28.3)	64(25.5)	392(29.0)	79(27.8)		
Surgery	738(39.1)	87(34.7)	545(40.3)	106(37.3)		
Obstetrics and gynecology	94(5.0)	11(4.4)	69(5.1)	14(4.9)		
Pediatrics	136(7.2)	12(4.8)	104(7.7)	20(7.0)		
Emergency	164(8.7)	23(9.2)	101(7.5)	40(14.1)		
Other	221(11.7)	54(21.5)	142(10.5)	25(8.8)		
Professional title, (%)					11.083	**0.026**^a^
Primary	894(47.4)	141(56.2)	612(45.2)	141(49.6)		
Intermediate	914(48.4)	100(39.8)	683(50.5)	131(46.1)		
Senior	80(4.2)	10(4.0)	58(4.3)	12(4.2)		
Employment type, (%)					12.495	**0.002**^a^
Formal employee	409(21.7)	33(13.1)	313(23.1)	63(22.2)		
Contract employee	1479(78.3)	218(86.9)	1040(76.9)	221(77.8)		
Number of vacation days, (%)					19.991	**0.003**^a^
0	208(11.0)	21(8.4)	144(10.6)	43(15.1)		
1–14	1122(59.4)	142(56.6)	816(60.3)	164(57.7)		
15–30	291(15.4)	36(14.3)	205(15.2)	50(17.6)		
> 30	267(14.1)	52(20.7)	188(13.9)	27(9.5)		
Work hours per week, (%)					26.131	** < 0.001**^a^
≤ 40	166(8.8)	30(12.0)	120(8.9)	16(5.6)		
41–50	1283(68.0)	162(64.5)	927(68.5)	194(68.3)		
51–60	262(13.9)	31(12.4)	201(14.9)	30(10.6)		
> 60	177(9.4)	28(11.2)	105(7.8)	44(15.5)		
FFWV level, (%)					53.069	** < 0.001**^a^
Low	138(7.3)	43(17.1)	83(6.1)	12(4.2)		
Medium	375(19.9)	57(22.7)	247(18.3)	71(25.0)		
High	1375(72.8)	151(60.2)	1023(75.6)	201(70.8)		
Scores of depressive symptoms, (mean ± SD)	9.19 ± 5.40	6.51 ± 4.49	9.20 ± 5.30	11.52 ± 5.56	116.329	** < 0.001**^b^
Depressive symptoms, (%)					83.550	** < 0.001**^a^
Yes	866(45.9)	61(24.3)	624(46.1)	181(63.7)		
No	1022(54.1)	190(75.7)	729(53.9)	103(26.3)		

*Abbreviations*: *WPV* Workplace Violence, *FFWV* Fear of future workplace violence, *CNY* Chinese Yuan, 1USD≈6.96 CNY ( 2020.08 exchange rate), *SD *Standard deviations. ^a^*P*-values are derived from χ^2^ tests, ^b^*P*-values were derived from Kruskal–Wallis test

There were statistically significant differences in age, sex, marital status, living condition, monthly income, number of children, department, professional title, employment status, number of vacation days, work hours per week, level of FFWV, and depressive symptoms among the groups (*p* < 0.05).

### Scores of depressive symptoms according to levels of FFWV

Table 2 shows the scores of depressive symptoms and the levels of FFWV among the groups of nurses with different WPV experiences. Among those in both no aggression group and non-physical violence group, there were significant differences in the levels of FFWV and the scores of depressive symptoms (*p* < 0.05). Between those in the physical violence group, there were no significant differences in the levels of FFWV and the scores of depressive symptoms.

**Table 2:** Distribution of scores of depressive symptoms according to FFWV between groups

Groups	Level of FFWV	Depressive symptoms (mean ± SD)	*F*	*P*
No aggression			4.019	**0.019**
	Low	4.77 ± 3.15		
	Medium	6.86 ± 4.02		
	High	6.88 ± 4.87		
Non-physical violence			13.577	** < 0.001**
	Low	6.87 ± 5.07		
	Medium	8.41 ± 5.08		
	High	9.58 ± 5.32		
Physical violence			0.252	0.778
	Low	12.58 ± 5.65		
	Medium	11.59 ± 5.57		
	High	11.52 ± 5.56		

*Abbreviations: SD* Standard deviations

### Multiple logistic regression analysis of FFWV and depressive symptoms

Table 3 shows the associations between FFWV and depressive symptoms among the nurses. After controlling for confounding factors, the results of the multiple logistic regression analysis revealed that among the no aggression group, a high level of FFWV was associated with higher levels of depressive symptoms (odds ratio [OR] = 3.269, 95% confidence interval [CI]: 1.102–9.695). Among the non-physical violence group, a high level of FFWV was associated with higher levels of depressive symptoms (OR = 2.338, 95% CI: 1.385–3.945). Among the physical violence group, there was no significant correlation between FFWV and depressive symptoms.

**Table 3:** Multiple logistic regression analysis on the association between fear of future workplace violence and depressive symptoms among groups

Variables	Model 1		Model 2		Model 3	
	OR	95% CI	OR	95% CI	OR	95% CI
No aggression						
FFWV						
Low (ref.)						
Medium	2.966	0.990–8.882	3.100	0.976–9.850	2.897	0.870–9.647
High	2.739*	1.008–7.445	2.928*	1.022–8.388	3.269*	1.102–9.695
Non-physical violence						
FFWV						
Low (ref.)						
Medium	1.716	0.996–2.956	1.554	0.891–2.711	1.673	0.946–2.960
High	2.523***	1.537–4.144	2.202**	1.322–3.668	2.338**	1.385–3.945
Physical violence						
FFWV						
Low (ref.)						
Medium	0.696	0.172–2.815	0.733	0.168–3.192	0.829	0.176–3.914
High	0.537	0.141–2.044	0.612	0.145–2.581	0.678	0.148–3.102

*OR* Odds ratio, *CI* Confidence interval, *FFWV* Fear of future workplace violence

Model 1: crude model. Model 2: adjusted for sex, age, marital status, living condition, education level, monthly income and number of children. Model 3: adjusted for sex, age, marital status, living condition, education level, monthly income, number of children, department, professional status, employ type

*p < 0.05, **p < 0.01, ***p < 0.001

## Discussion

This is the first study to investigate the association between FFWV and depressive symptoms among nurses in China. In this study, the prevalence of depressive symptoms among nurses who had experienced WPV was higher (physical violence group: 63.7%; non-physical violence group: 46.1%) than among those who had no experience of WPV (no aggression group: 24.3%). The positive association between WPV and depressive symptoms among nurses has been assessed in many previous studies [3, 10]. WPV has negative effects on nurses’ health, such as emotional exhaustion and poor quality of sleep [10], which could lead nurses to experience more severe depressive symptoms [29]. In addition, in the current study, the prevalence of depressive symptoms among nurses who had experienced physical violence was higher than among those who had experienced non-physical violence (63.7% vs 46.1%, respectively). This result is consistent with a previous study that found that medical staff who experienced physical violence were more likely to develop depressive symptoms than those who had not experienced physical violence [3].

In this study, nurses who had experienced non-physical violence had the highest level of FFWV (75.6%), followed by nurses who had experienced physical violence (70.8%), and nurses who had not experienced WPV (60.2%). This finding indicates that the experience of WPV can generate FFWV [15]. Furthermore, nurses who had not experienced WPV also had a relatively high level of FFWV. Due to the high incidence of WPV against nurses, they frequently observe news reports about WPV against medical staff or personally witness WPV, which may lead them to develop FFWV. In our study, nurses in the physical violence group had less FFWV than those in the non-physical violence group. There are two possible reasons for this phenomenon. First, physical violence is one of the worst types of WPV and typically has the greatest negative impact on nurses’ health. When nurses are victims of physical violence, hospital leaders, colleagues, and family members often provide material compensation and emotional support [30]. Such social support could help nurses to reduce their FFWV [25]. Unlike physical violence, non-physical violence against nurses in the workplace is often ignored by hospital managers, peers, or family members due to nurses viewing it as “part of the job”; hence, the reporting rate for non-physical violence is very low [31]. The organizational culture of hospitals and nurses often exhibits zero tolerance for physical violence as opposed to non-physical violence [32]. However, non-physical violence is also a source of fear; nurses often describe it as a constant source of anxiety that inevitably forms part of the role [33]. Second, a previous study has found that WPV can enhance nurses’ awareness; nurses can learn to adopt preventive behaviors, improve their relevant work-related skills, and thus cultivate greater tenacity [34]. As such, nurses who experience physical violence may become more confident in preventing and coping with possible future WPV. However, nurses who have experienced non-physical violence may lack the experience and ability to cope with possible future physical violence. Therefore, they may have more FFWV than those who have experienced physical violence. These findings emphasize that there is a high level of FFWV among many nurses, especially among those who have experienced WPV.

Our findings showed that FFWV was positively associated with depressive symptoms among nurses in the no aggression group and in the non-physical violence group. Fear can affect individuals’ self-image and confidence, leading them to feel unsafe and hence experience negative psychological consequences [35]. Nurses with high levels of FFWV are more likely to engage in unnecessarily avoidant behaviors [36], which could lead to a lack of trust between them and their patients or colleagues, which could increase the social isolation of such nurses. This could eventually reduce their quality of life and foster depression [35]. Fu found that FFWV was positively associated with emotional exhaustion and cynicism among nurses [24], while another study showed that nurses with a high level of emotional exhaustion or cynicism were more likely to experience depressive symptoms [37].

In this study, we did not find a statistically significant association between FFWV and depressive symptoms among nurses in the physical violence group. This may be because nurses who had experienced physical violence were more likely to have greater psychological resilience and more confidence in being able to take action to prevent them from being future victims of WPV [38]; this could further help them to improve their work ability [39], and thus reduce the occurrence of unnecessary avoidance behavior in the workplace. In addition, nurses who experience physical violence are more likely to receive care and support [30], which helps reduce their social isolation, and eventually reduces the effects of FFWV on depressive symptoms [40]. Although we did not find a statistically significant association between FFWV and depressive symptoms among the nurses in the physical violence group, hospital management should also consider the extent of depressive symptoms in members of this group, because this group had the highest prevalence of depressive symptoms among the current sample of nurses. Further research is needed to determine the factors that influence depressive symptoms among nurses who experience physical violence.

### Limitations

There are several limitations to this study. First, there was a risk of recall bias, which could have led to inaccurate responses. Second, the causal relationship between FFWV and depressive symptoms could not be determined because this was a cross-sectional study. Third, the participants only included those from tertiary hospitals, which may limit the generalizability of our results to primary and secondary hospitals. Fourth, some variables such as the safety of workplace, and nurses’ coping strategies with WPV which may have effects on both FFWV and mental health were not investigated in the study. These variables will be considered in future studies. Last, this study was conducted during the COVID-19 epidemic, though the progress in containing the epidemic has been made and restrictions are gradually relaxed in China, the impact of the COVID-19 epidemic on nurses’ mental health needs to be considered [41].

## Conclusion

Nurses who had experienced physical violence had the highest prevalence of depressive symptoms and nurses with experience of non-physical violence had the highest level of FFWV. Nurses with a high level of FFWV were more likely to have greater levels of depressive symptoms in both the no aggression group and the non-physical violence group. There was no statistically significant association between FFWV and depressive symptoms among nurses in the physical violence group. The findings of this study could be used to help develop strategies to reduce depressive symptoms among nurses.

### Policy implications

Based on our findings, we suggest that hospital managers should: (1) pay more attention to depressive symptoms among nurses who have experienced WPV, and especially among those who have experienced physical violence; (2) consider FFWV when developing interventions to reduce depressive symptoms among nurses who have not been victims of aggression and among those who have experienced non-physical violence; (3) adopt effective measures to reduce the level of FFWV among nurses due to its negative effects on nurses’ health and work performance; (4) pay more attention to non-physical violence (verbal violence and psychological violence) against nurses, and provide better care and support for nurses who have experienced non-physical violence; and (5) enhance nurses’ training in relation to preventing WPV to increase their confidence and ability to cope with WPV.

## Data Availability

The datasets generated and/or analyzed during the current study are not publicly available due to agreements with participants who restricted data sharing but are available from the corresponding author on reasonable request.

## Abbreviations

ANOVA: Analysis of variance
FFWV: Fear of future workplace violence
WPV: Workplace violence
CNY: Chinese Yuan
USD: United States dollar
OR: Odds ratio
CI: Confidence interval
SD: Standard deviations
